# Preferences for Delivery of HIV Prevention Services Among Healthcare Users in South Africa: A Discrete Choice Experiment

**DOI:** 10.1007/s10461-024-04519-4

**Published:** 2024-10-01

**Authors:** Catherine Elizabeth Martin, Duane Blaauw, Pelisa Nongena, Glory Chidumwa, Siphokazi Dada, Samantha Jack, Vusile Butler, Saiqa Mullick

**Affiliations:** 1 Wits RHI, University of the Witwatersrand, Johannesburg, South Africa; 2 Faculty of Health Sciences, Centre for Health Policy, University of the Witwatersrand, Johannesburg, South Africa

**Keywords:** HIV prevention, PrEP, Service delivery model, Differentiated care, Discrete choice experiment

## Abstract

Progress has been made to scale oral pre-exposure prophylaxis (PrEP) as part of combination HIV prevention, with the WHO recommending differentiated, simplified and demedicalized approaches. This study explored user preferences for components of a PrEP service delivery package, through a discrete choice experiment (DCE) among 307 people accessing primary healthcare services in South Africa between November 2022 and February 2023. Attributes included were: Source of information about HIV prevention and PrEP; Site for PrEP initiation and follow-up; Frequency of follow-up; PrEP pick-up point; HIV testing whilst using PrEP; Contact between appointments. A D-efficient DCE design was created with 16 choice sets in 2 blocks. DCE data were analysed using generalised multinomial logistic models. Compared to printed materials, participants preferred getting information about PrEP online (aOR = 7.73, 95% CI = 5.13–11.66) and through WhatsApp (aOR = 2.23, 95% CI = 0.98–5.55). PrEP initiation at a pharmacy or mobile clinic was valued equally to initiating PrEP at a clinic, but a community pop-up site was less preferred (aOR = 0.46, 95% CI = 0.33–0.64). There was a preference for 6-monthly over 3-monthly follow-up (aOR = 11.88, 95% CI = 5.44–25.94). Participants preferred collecting PrEP from a pharmacy (aOR = 5.02, 95% CI = 3.45–7.31), through home delivery (aOR = 2.18, 95% CI = 1.26–3.78) and from a vending machine (aOR = 1.43, 95% CI = 1.02–1.99) relative to where they initiated PrEP. Participants also preferred HIV self-testing over a healthcare provider test (aOR = 5.57, 95% CI = 3.72, 8.36). WhatsApp or Facebook groups (aOR = 4.12, 95% CI = 3.00–5.67), monthly phone calls (aOR = 2.84, 95% CI = 1.73–4.67) and weekly messages (aOR = 1.47, 95% CI = 1.10–1.97) were preferred contact between appointments, relative to no contact. To meet users’ preferences, there is a need to expand decentralised and self-led HIV prevention services.

**Supplementary Information:**

The online version contains supplementary material available at 10.1007/s10461-024-04519-4.

## Introduction

Substantial progress has been made in HIV prevention efforts through the scale up of oral pre-exposure prophylaxis (PrEP) as part of a combination package of HIV prevention services in South Africa, with over 1,4 million people initiated at over 4000 health facilities as of February 2024 [1]. However, barriers to service access as well as effective use of PrEP, particularly for young people, remain. Oral PrEP demonstration projects have reported high initial uptake, but challenges with continued use [2–5]. Individual barriers include stigma, pill burden and PrEP side effects [6], with service delivery barriers including school and work scheduling conflicts, distance from service delivery sites, clinic operating hours, long waiting times and healthcare provider attitudes [6–8]. As outlined in the World Health Organization’s (WHO) implementation guidance [9], differentiated, simplified and demedicalized PrEP services may make services more acceptable and accessible, whilst also supporting PrEP uptake, persistence and effective use. They outline four building blocks to PrEP service delivery: *where* (service location), *who* (service provider), *when* (service frequency) and *what* (service package) [9].

PrEP in South Africa is currently provided as part of a package of HIV prevention services, and aside from a small number of donor–funded projects implementing out of facility models [10–12], is predominantly provided at nurse-led public sector primary care health facilities. Delivery of oral PrEP through community-based models in South Africa has been shown to be feasible and acceptable [10, 11, 13, 14], with benefits including convenience, youth-friendliness, reduction in transportation costs, and increased trust and ownership of the services provided [10]. Pharmacy PrEP delivery models have also been described and reported to be acceptable among end users and providers [15], although implementation data from the region is limited. PrEP users have highlighted the benefit to PrEP uptake that having services available in more convenient community-based locations could achieve [6], with research also suggesting acceptability among healthcare providers [9]. New PrEP methods, such as the monthly dapivirine vaginal ring (DVR), two-monthly injectable cabotegravir (CAB-LA) and 6-monthly injectable lenacapavir may change the HIV prevention landscape and provide further opportunity for different models of PrEP delivery.

Although not yet incorporated into HIV testing guidelines in South Africa [16], the use of HIV self-testing in PrEP programmes is supported by the WHO and could also support differentiated service delivery approaches, reduce clinic visits and improve privacy and convenience [9]. Other service package components which could enhance uptake and effective use of PrEP include the provision of accurate, accessible information and messaging about PrEP [6, 17]; flexibility in timing of PrEP refills [17]; and ongoing support for continued PrEP use by healthcare providers and peers [6, 17].

As differentiated PrEP service delivery models are developed and implemented, it is important to understand end user preferences, in order to ensure that investments are made in delivery models most likely to reach and be acceptable to those who need them. Discrete Choice Experiments (DCE) have been used in health research as a quantitative method to evaluate user preferences for health products and service delivery models [18, 19]. DCEs evaluate the key features or attributes of a product or service which are of value to an individual. Potential users are asked to choose between different products or services made up of carefully selected combinations of these attributes. From those choices the analyst can determine the value and relative importance of each attribute, and identify the combinations most likely to influence decision making.

Through a DCE aligned to the WHO building blocks of a differentiated PrEP service delivery package, this study aimed to explore healthcare users’ preferences for key components of a PrEP service delivery package.

## Methods

This study was a cross-sectional survey and DCE conducted between November 2022 and February 2023 among HIV-negative men and women accessing sexual and reproductive health and related services at primary care clinics in South Africa.

### Study Setting

The study was conducted in eight public primary care clinics in four geographical areas in three provinces in South Africa. These study sites are part of an ongoing implementation science study conducted by Wits RHI since 2018, which aims to generate evidence around the introduction and integration of PrEP as part of combination HIV prevention services in South Africa, and through which oral PrEP has been introduced. The sites comprise two clinics in a peri-urban area in a residential township on the outskirts of Gqeberha in the Eastern Cape; two clinics in the rural town of Mthatha in the Eastern Cape, two clinics in an urban area of eThekwini in Kwa-Zulu Natal and two clinics in a peri-urban area of Tshwane in Gauteng. All are in areas with a high burden of HIV and are currently providing nurse-initiated oral PrEP as part of a package of HIV prevention services at these sites and through a linked mobile clinic in each area.

### Development of the Discrete Choice Experiment

**Fig. 1:**
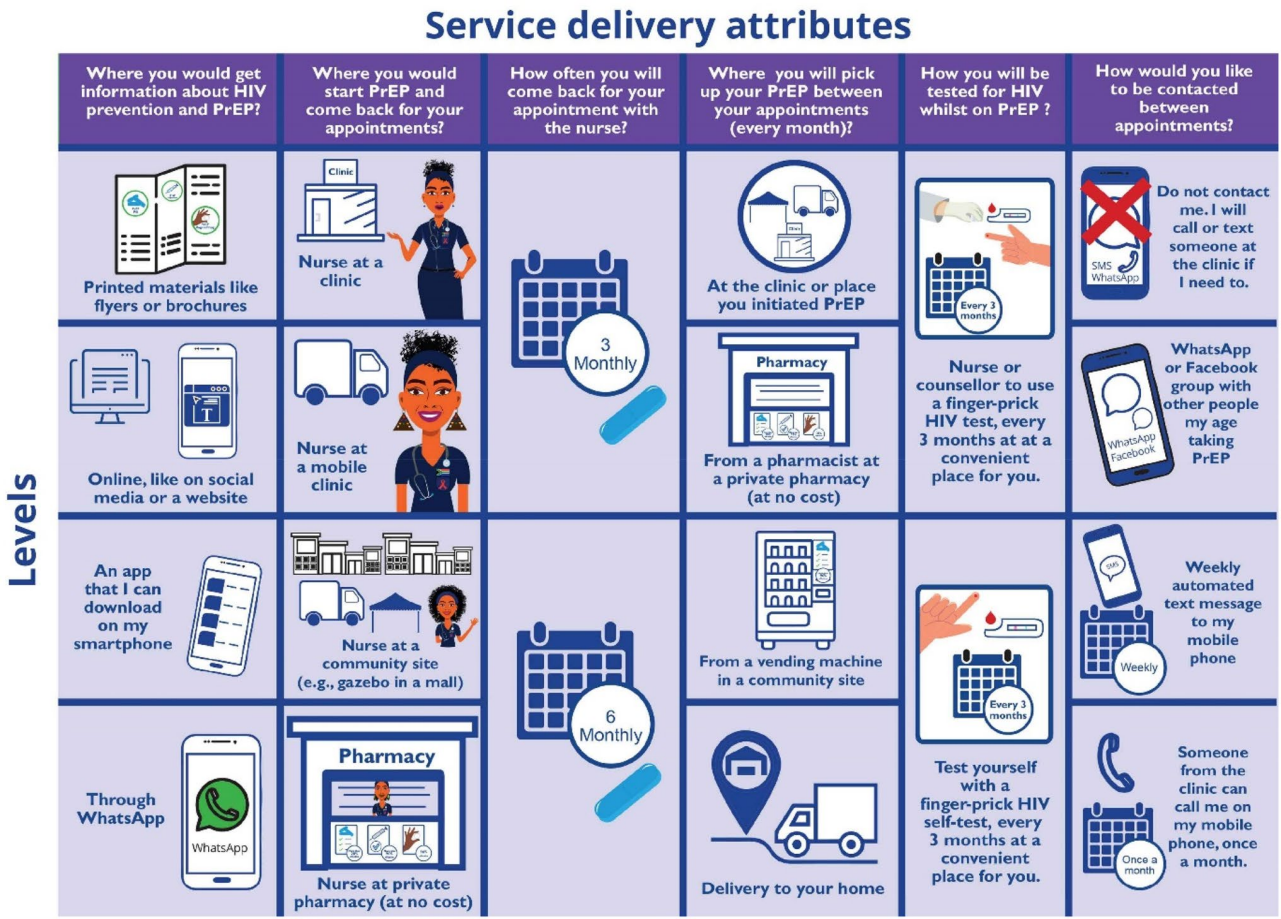
PrEP service delivery model attributes and levels included in the discrete choice experiment

Literature review, complemented by formative research conducted through workshops and in-depth interviews with twelve adolescent girls and young women, and nine healthcare providers informed the selection of the PrEP service delivery attributes and levels. Through iterative review by the broader study team, which included technical experts, programme implementers and young people, a final design was developed, aligned to the WHO building blocks of PrEP service delivery. Attributes included were: (1) Source of information about HIV prevention and PrEP; (2) PrEP initiation site and clinical follow-up; (3) Frequency of follow-up appointments; (4) PrEP pick up point between clinical appointments; (5) HIV testing method whilst using PrEP; and (6) Contact between appointments for general support for PrEP use. Images were developed and included as a visual aid for each attribute level. Extensive piloting of the tool was conducted prior to implementation. Following an initial pilot, the tool was redesigned and simplified. The revised tool was then tested during a second pilot phase to ensure that it was comprehensible to the target audience. The final attributes, levels and images are presented in Fig. 1.

**Fig. 2:**
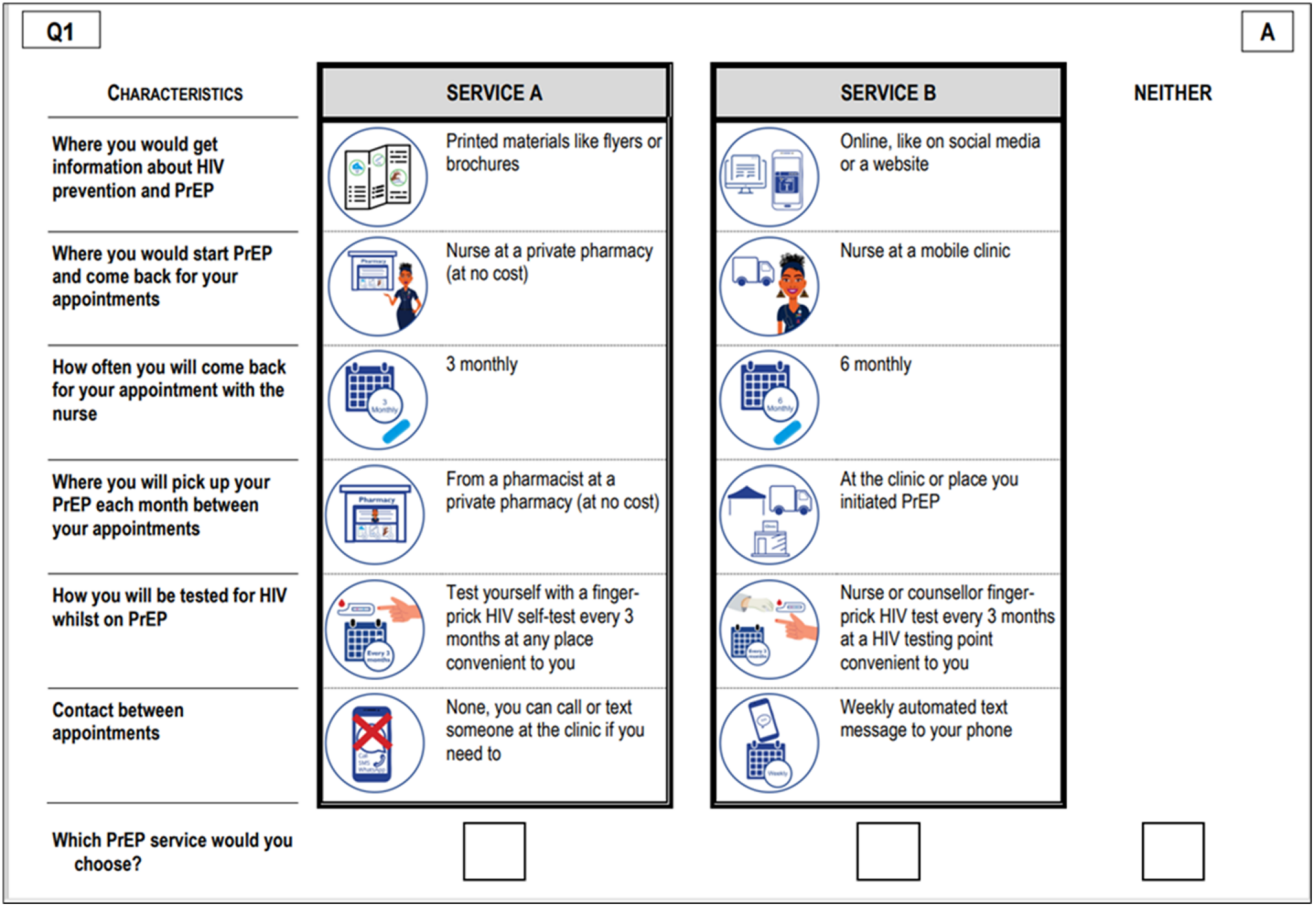
Example of the discrete choice experiment choice set

We used a generic DCE design with 16 choice sets. Each choice set had three alternatives, with participants choosing either “Service A” or “Service B”, or an opt-out choice for those who preferred neither service option (see example in Fig. 2). The construction of the choice sets was done using a D-efficient experimental design in the Ngene software programme [20] with priors obtained from the pilot study. To decrease the number of choice sets answered by each participant, the full design was split into two blocks of eight questions, and two different orderings of each block were then created to minimise ordering effects. Participants were randomly assigned to the different blocks and orders during data collection.

### Sampling and Recruitment

Eligible participants were HIV-negative (self-reported) males or females aged 15 years and older who were accessing sexual and reproductive health or related services at a study site. Study fieldworkers consecutively approached clients accessing services at study sites, provided them with information about the study and screened them for eligibility. After providing written informed consent for study participation, they completed a structured survey and the DCE.

### Data Collection

Interviews took approximately 45 min to complete and were conducted in English, in-person at the study sites by trained fieldworkers. Data were captured directly on password-protected tablet computers using REDCap electronic data capture tools hosted by the University of the Witwatersrand [21, 22]. The survey comprised of three sections: socio-demographics, the DCE choice sets and socio-behavioural characteristics, which included questions on sexual and reproductive health and PrEP method preferences. For the DCE, interviewers began by explaining the choice task and providing an overview of each attribute using a printed card as a visual aid. The eight DCE choice sets were also presented to participants on individual printed cards, with the responses captured on the tablet.

### Data Analysis

Descriptive statistics were used to describe the participants sociodemographic and behavioural characteristics. We utilised different logistic regression methods to assess the relative importance of service delivery attributes in the DCE. The conditional logit model is the basic workhorse of DCE analysis [23]. The mixed logit further allows for preference heterogeneity and adjusts for the panel nature of the data [24]. The generalised multinomial logit (G-MNL) incorporates both preference and scale heterogeneity which may be important in comparing sub-groups with different variances [25]. Only the G-MNL results are shown in this paper. Adjusted odds ratios (aOR) and 95% confidence intervals (95% CI) were reported. We compared the preferences of different subgroups through stratified models, including by gender, age group, educational level, employment status, province and previous PrEP use. The statistical significance of subgroup differences were evaluated in pooled regression models which included interaction terms between the subgroup and design variables. For parsimony, only the more significant interactions (p value less than 0.1) were retained in the final interaction model. Data manipulation and general analyses were done in Stata v17 while Limdep/Nlogit v6 [26] was used for the choice modelling.

### Ethical Considerations

This study was approved by the Human Research Ethics Committees at the University of the Witwatersrand (M210849), and the Research Ethics Committee of the World Health Organization. In addition, approval was provided from the local department of health and the research committees of the provinces in which the research was conducted. Written informed consent (or parental consent and assent in the case of minors less than 18 years of age) was provided by participants before participation in study activities. In the case of minor participants, parental consent was obtained in-person from parents who were present at the facility at the time of recruitment. All participants were reimbursed ZAR100 ($5.50) for their time and effort taken to participate in the study.

## Results

A total of 372 participants were screened, of whom 345 (93%) were eligible and 312 (90%) of these agreed to participate, 114 males and 198 females. Reasons for non-eligibility included being HIV positive (n = 7) and minors unable to provide parental consent for study participation (n = 20). Data for 5 participants were excluded because they chose the same alternative in all eight choice tasks indicating that they were not properly considering the differing attribute levels. That left a final analysis sample of 307, 113 males (36.8%) and 194 females (63.2%). The median age was 23 years (Inter-quartile range [IQR]: 21–31). Of the 307 participants, 43% were adolescent girls or young women aged 15–24 years (n = 132), 80% (n = 246) had completed secondary school or higher and 61% were unemployed (n = 187). In addition, 171 participants (55.7%) had used oral PrEP before while 49% (n = 151) were currently using oral PrEP. Table 1 shows the sociodemographic and sexual behavioural characteristics of study participants by sex.

**Table 1:** Socio-demographic and sexual behavioural characteristics of study participants by sex, N = 307

	Male n = 113 (36.8%)		Female n = 194 (63.2%)		Total N = 307 (100%)	
	No	%	No	%	No	%
Enrolment site						
Mthatha	29	25.7%	46	23.7%	75	24.4%
Gqeberha	28	24.8%	47	24.2%	75	24.4%
eThekwini	28	24.8%	49	25.3%	77	25.1%
Tshwane	28	24.8%	52	26.8%	80	26.1%
Age group (years)						
15–24	41	36.3%	132	68.0%	173	56.4%
25 +	72	63.7%	62	32.0%	134	43.6%
Highest level of education						
Less than secondary school	24	21.2%	37	19.1%	61	19.9%
Completed secondary school	60	53.1%	114	58.8%	174	56.7%
Higher than secondary school	29	25.7%	43	22.2%	72	23.5%
Employment status						
Employed	64	56.6%	56	28.9%	120	39.1%
Unemployed	49	43.4%	138	71.1%	187	60.9%
Number of sexual partners						
No sexual partners	2	1.8%	14	7.2%	16	5.2%
One sexual partner	54	47.8%	149	76.8%	203	66.1%
More than one sexual partner	48	42.5%	25	12.9%	73	23.8%
Did not wish to answer	9	8.0%	6	3.1%	15	4.9%
Type of contraceptives used						
Oral contraceptives			17	8.8%		
Injectable contraceptives			95	49.0%		
IUD/IUS/Implant			22	11.3%		
Male/female condoms			12	6.2%		
None			18	9.3%		
Missing			30	15.5%		
Currently pregnant			17	8.8%		
Currently breastfeeding			18	9.3%		
Ever used oral PrEP before	62	54.9%	109	56.2%	171	55.7%
Currently using oral PrEP	58	51.3%	93	47.9%	151	49.2%

Table 2 shows the results from the G-MNL DCE analysis of the determinants of service delivery preferences. Participants were nearly eight times more likely to choose getting information about PrEP online (aOR = 7.73, 95% CI: 5.13; 11.66, p < 0.001) and twice as likely to choose getting information on WhatsApp over printed material, although this was marginally significant. Participants strongly preferred initiating PrEP at a clinic compared to at a pop-up community site (e.g., gazebo in a mall) (aOR = 0.46, 95% CI: 0.33; 0.64, p < 0.001), but did accept initiating PrEP at a mobile clinic (aOR = 1.30, 95% CI: 1.01; 1.69, p = 0.043). There was strong evidence of preference for 6-monthly follow-up appointments over 3-monthly follow-up appointments (aOR = 11.88, 95% CI: 5.44; 25.94, p < 0.001).

**Table 2:** Generalised multinomial logistic (G-MNL) regression for determinants of service delivery preferences

Attribute	Level	Parameter mean				Parameter SD			
		OR	95% CI	p value		OR	95% CI	p value	
Get information about PrEP	Printed materials	–				–			
	Online	7.73	[5.13; 11.66]	< 0.001	***	1.45	[1.00; 2.08]	0.048	*
	Mobile application	1.23	[0.90; 1.66]	0.192		1.94	[1.49; 2.52]	< 0.001	***
	WhatsApp	2.33	[0.98; 5.55]	0.056		1.97	[1.44; 2.69]	< 0.001	***
Start PrEP	Nurse at clinic								
	Nurse at mobile	1.30	[1.01; 1.69]	0.043	*	1.64	[1.09; 2.47]	0.018	*
	Nurse at community site	0.46	[0.33; 0.64]	< 0.001	***	2.75	[2.01; 3.76]	< 0.001	***
	Nurse at private pharmacy	0.79	[0.33; 1.91]	0.602		1.24	[0.86; 1.81]	0.251	
Frequency of follow-up appointments	3 monthly								
	6 monthly	11.88	[5.44; 25.94]	< 0.001	***	1.92	[1.50; 2.45]	< 0.001	***
Collect PrEP between appointments	Where you initiated PrEP								
	Pharmacist at private pharmacy	5.02	[3.45; 7.31]	< 0.001	***	2.60	[1.92; 3.53]	< 0.001	***
	Community vending machine	1.43	[1.02; 1.99]	0.036	*	1.93	[1.51; 2.46]	< 0.001	***
	Home delivery	2.18	[1.26; 3.78]	0.006	**	4.37	[3.04; 6.27]	< 0.001	***
3 monthly HIV testing while on PrEP	Healthcare provider HIV test								
	HIV self-test	5.57	[3.72; 8.36]	< 0.001	***	1.66	[1.35; 2.04]	< 0.001	***
Contact between appointments	None								
	WhatsApp / Facebook group	4.12	[3.00; 5.67]	< 0.001	***	1.16	[0.78; 1.73]	0.468	
	Weekly SMS message	1.47	[1.10; 1.97]	0.010	**	1.47	[1.06; 2.03]	0.020	*
	Monthly phone call from clinic	2.84	[1.73; 4.67]	< 0.001	***	1.15	[0.80; 1.66]	0.454	
Scale (Tau)		0.07	[− 0.19; 0.33]	0.6032					
n	2456								
LL	− 1759.2								
Pseudo R^2^	0.348								

*p < 0.05, **p < 0.01, ***p < 0.001

Participants were more enthusiastic about decentralised visits to collect PrEP after initiation. The strongest preference was to collect PrEP from a pharmacist at a private pharmacy (aOR = 5.02, 95% CI: 3.45; 7.31, p < 0.001) rather than where they initiated PrEP, followed by home delivery (aOR = 2.18, 95% CI: 1.26; 3.78, p = 0.006) and a community vending machine (aOR = 1.43, 95% CI: 1.02; 1.99, p = 0.036). Participants preferred an HIV self-test over a healthcare provider test (aOR = 5.57, 95% CI: 3.72; 8.36, p < 0.001). Participants also clearly valued some contact between appointments compared to none, favouring WhatsApp or Facebook groups (aOR = 4.12, 95% CI: 3.00; 5.67, p < 0.001) over monthly phone calls from the clinic (aOR = 2.84, 95% CI: 1.73; 4.67, p < 0.001) and weekly SMS messages (aOR = 1.47, 95% CI: 1.10; 1.97, p = 0.010). There was significant heterogeneity within the group for most of these preferences, as indicated by the statistically significant parameter standard deviations (SD) in Table 2.

**Fig. 3:**
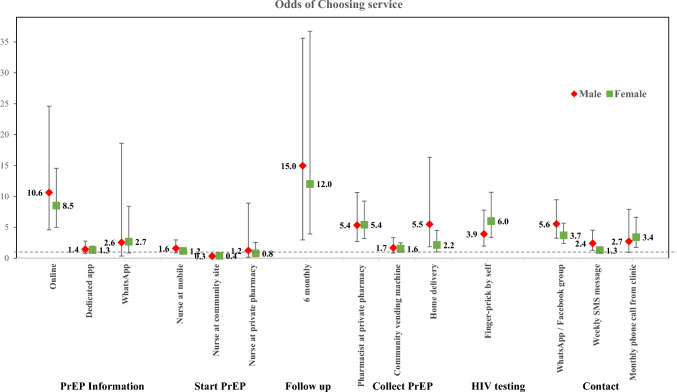
Generalised multinomial logistic (G-MNL) logistic regression for determinants of service delivery preferences by sex

**Fig. 4:**
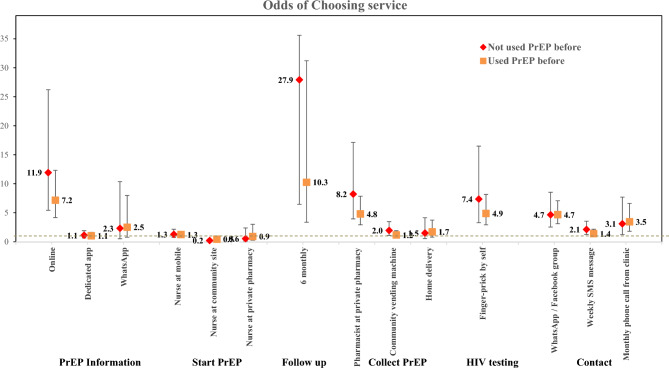
Generalised multinomial logistic (G-MNL) logistic regression for determinants of service delivery preferences by PrEP use

Stratified analyses by sex and previous PrEP use showed no marked differences in service delivery preferences between the subgroups (See Figs. 3 and 4). However, there were a number of statistically significant differences between subgroups in the formal pooled interaction models (Supplementary Table 1). Males were less enthusiastic about a dedicated app for getting information than females, but did prefer the WhatsApp group contact more. Respondents older than 25 years of age rated the weekly SMS contact more than those under 25 years. Users from KwaZulu-Natal and the Eastern Cape provinces had higher preferences for collecting PrEP from vending machines or delivery at home, than those from Gauteng. The only significant difference related to previous PrEP experience was that those who had used PrEP before were less enthusiastic about HIV self-testing.

## Discussion

This study among young adult male and female healthcare users in South Africa examines PrEP service delivery preferences using a DCE and provides insights which can inform the development and implementation of differentiated PrEP service delivery models. Healthcare users indicated a preference for information about PrEP to be available through online materials and the use of WhatsApp. PrEP initiation at a pop-up community site was less preferred to a fixed clinic, although there was a preference for collection of PrEP between clinic visits at a private pharmacy, community vending machine or through home delivery. There was a strong preference for follow up appointments to be 6-monthly compared to 3-monthly, and for the use of self-screening to test for HIV whilst using PrEP. A WhatsApp or Facebook group with peers or a monthly phone call or SMS from the clinic was preferred over no contact between visits. We found some heterogeneity in preferences, including by sex, age, province and previous experience of PrEP.

The growing demand for health information to be delivered through online platforms, social media and WhatsApp is not surprising given the advances in technology and high rates of internet penetration in South Africa [27]. Existing platforms such as the South African Department of Health’s BWise [28] and MyPrEP.co.za [29] websites offer opportunities to meet this preference. Digital health technology has the additional potential to improve individual assessment of need for PrEP and linkage to services [9], with benefits including accessibility, privacy, convenience, autonomy and reduced stigma [30–32]. Our findings further suggest that online and social media platforms may be a preferred modality for supporting ongoing PrEP use and engagement with care between visits, although further research is required to determine the effectiveness of these interventions.

Our results indicate that in our context, community-based PrEP initiation sites may not hold substantially greater appeal than fixed clinics, and that people are likely to prefer clinical services to be offered in a more structured environment than at pop-up sites or gazebos. This finding is supported by healthcare providers and could be due to the need to ensure that services can be provided discretely and confidentially. Clinic environments may also be more familiar to healthcare users, with an understanding that in the South African context comprehensive primary care services, including HIV prevention, contraception, sexually transmitted infection (STI) and related services would be offered free of charge [33]. Although our findings showed only minor differences in preference between women and men, a study among South African youth found a mixed preference for PrEP service delivery sites, with women preferring clinics over pharmacies, male sex workers preferring community sites and men who have sex with men preferring pharmacies and clinics over community sites [34]. It is also plausible that the factors influencing PrEP delivery site preference may be related to attributes other than the location, such as attitudes of healthcare workers and anticipated costs [6].

The strong preferences for PrEP pick-ups through pharmacies, community vending machines and home deliveries, indicates a preference for more decentralised and de-medicalised options for continued PrEP access. These options may overcome some of the cited barriers to ongoing PrEP use, such as distance or transport requirements to get to clinics or not having enough time [8, 35]. Our findings suggest that different models may be preferred in different settings. These models hold potential for PrEP delivery in South Africa, given the well-established Chronic Medicine Dispensing and Distribution (CCMDD) programme that has been implemented for chronic medication pick-ups in South Africa [36]. This programme provides chronic medication (including antiretroviral treatment [ART]) and oral contraceptive refills for stable patients at community-based pick-up points, including a network of independent pharmacies. The programme had over 3.2 million patients registered as of October 2021 [37] with high acceptability reported among healthcare providers and patients, citing benefits including convenience, accessibility and flexibility [38]. Pilot studies examining the delivery of PrEP services through private pharmacies in Kenya have reported the feasibility and acceptability of these models [39]. Although published data on PrEP delivery through private pharmacies in South Africa is scarce, formative work has highlighted the potential acceptability of this model with benefits perceived to include privacy, confidentiality, and speed [33, 39].

It is interesting to note the preference for home delivery of PrEP, a model of health service which gained traction for delivery of medicines during the COVID pandemic [40, 41], and which has shown promise for the delivery of ART in South Africa [42]. Although there is little published literature on home delivery of PrEP in South Africa, or the region, there are some pilot studies describing home-based PrEP services among men who have sex with men, complemented by self-collection of specimens which were laboratory tested [43, 44]. However, concerns have been raised about the costs, feasibility, and sustainability of such models in limited resource settings [45], and further research on these models are needed.

The desire for less frequent follow-up visits is not surprising, given that clinic access has been cited as a barrier to PrEP use in prior literature [6, 46]. Our findings also highlight the willingness that people may have to use HIV self-tests, particularly among those who have not accessed PrEP before, which could improve access to testing and PrEP, reduce the need for frequent clinic follow up visits and enhance self-led HIV prevention. A clinical trial in Kenya found that 6-monthly PrEP dispensing with the use of HIV self-testing reduced clinic visits and did not compromise HIV testing, retention or adherence [47].

It is notable that this study focused broadly on PrEP, and that some of the attributes evaluated may not currently be feasible for the newer PrEP methods that are being introduced through implementation studies in South Africa. For example, after the initiation doses, CAB-LA is required to be given 2-monthly and may not be easily amenable to multi-month provision. It would also not currently be feasible to provide an injectable method through a vending machine or home delivery, in the absence of self-administered injectable methods or without the support of a healthcare provider. Further evaluation of the use of HIV self-testing among people initiating and using CAB-LA is also required, given the potential challenges with diagnosing acute HIV that have been raised [48]. While it remains to be seen if these service delivery preferences could ultimately influence choice and use of the newer PrEP methods, placing user preferences at the centre of new product introduction and development of service delivery models is essential, so as to inform the best approaches for communication of new information and the development of delivery models, which meet their needs and preferences.

## Limitations

This study had limitations which should be noted. As current legislation in South Africa only allows for the prescription of PrEP by medically trained healthcare providers, the preference for delivery of PrEP by other health cadres was not explored. Participants were already accessing health services, and this study may not therefore reflect the preferences of individuals who are not engaged or able to easily access health services. Our findings may not be generalizable to other populations accessing PrEP, such as men who have sex with men, people who inject drugs or sex workers, who may have different service delivery preferences. Findings are also limited to the context in which this study was conducted. DCEs evaluate stated preferences rather than revealed preferences, although there is some evidence that DCEs have reasonable external validity [49].

## Conclusions

The findings of this study support the need to expand decentralised and self-led HIV prevention services to meet healthcare users’ preferences, including the incorporation of digital health technology, out of facility PrEP pick-up points, longer follow up periods between clinic visits and the use of HIV self-testing within HIV prevention services. Tailoring PrEP service delivery models to users’ preferences may enhance uptake and facilitate effective use.

## Supplementary Information

Below is the link to the electronic supplementary material.

Supplementary file1 (DOCX 20 KB)

## Data Availability

The data that support the findings of this study are available from the corresponding author upon request.
