# Supplementary material for: Preferences for Delivery of HIV Prevention Services Among Healthcare Users in South Africa: A Discrete Choice Experiment
Source: AIDS Behav. 2024 Oct 1;29(1):331–41. doi: 10.1007/s10461-024-04519-4 (PMC11739183; doi:10.1007/s10461-024-04519-4)
Supplement: Supplementary file 1 — Supplementary file1 (DOCX 20 KB) [file 10461_2024_4519_MOESM1_ESM.docx]

**Supplementary Table 1: Generalised multinomial logistic (G-MNL) model for impact of socio-demographic sub-groups on service preferences**

| **Attribute** |  | **Mean** | | | |  | **SD** | | | |
| --- | --- | --- | --- | --- | --- | --- | --- | --- | --- | --- |
|  |  | **OR** | **95%CI** | **p value** |  |  | **OR** | **95%CI** | **p value** |  |
| Get information about PrEP | Printed materials | — |  |  |  |  | — |  |  |  |
|  | Online | **8.16** | **[4.55; 14.62]** | **<0.001** | ******* |  | 1.45 | [0.99; 2.12] | 0.053 |  |
|  | Dedicated app | 1.20 | [0.88; 1.65] | 0.248 |  |  | **2.12** | **[1.65; 2.73]** | **<0.001** | ******* |
|  | WhatsApp | **2.30** | **[0.97; 5.46]** | **0.060** |  |  | **1.75** | **[1.23; 2.47]** | **0.002** | ****** |
| Start PrEP | Nurse at clinic |  |  |  |  |  |  |  |  |  |
|  | Nurse at mobile | 1.28 | [1.; 1.65] | 0.050 |  |  | **1.50** | **[0.99; 2.27]** | **0.056** |  |
|  | Nurse at community site | **0.40** | **[0.26; 0.6]** | **<0.001** | ******* |  | **2.78** | **[2.04; 3.8]** | **<0.001** | ******* |
|  | Nurse at private pharmacy | 0.77 | [0.32; 1.85] | 0.555 |  |  | 1.33 | [0.88; 2.01] | 0.180 |  |
| Frequency of follow-up appointments | 3 monthly |  |  |  |  |  |  |  |  |  |
|  | 6 monthly | **12.92** | **[4.77; 34.99]** | **<0.001** | ******* |  | **1.99** | **[1.56; 2.53]** | **<0.001** | ******* |
| Collect PrEP between appointments | Where you initiated PrEP |  |  |  |  |  |  |  |  |  |
|  | Pharmacist at private pharmacy | **4.93** | **[2.88; 8.44]** | **<0.001** | ******* |  | **2.39** | **[1.75; 3.26]** | **<0.001** | ******* |
|  | Community vending machine | 1.41 | [1.01; 1.96] | 0.044 | ***** |  | **1.77** | **[1.39; 2.27]** | **<0.001** | ******* |
|  | Home delivery | **2.47** | **[1.32; 4.61]** | **0.005** | ****** |  | **4.22** | **[2.91; 6.12]** | **<0.001** | ******* |
| 3 monthly HIV testing while on PrEP | Finger-prick by nurse |  |  |  |  |  |  |  |  |  |
|  | Finger-prick by self | **6.73** | **[3.61; 12.57]** | **<0.001** | ******* |  | **1.55** | **[1.25; 1.93]** | **<0.001** | ******* |
| Contact between appointments | None |  |  |  |  |  |  |  |  |  |
|  | WhatsApp / Facebook group | **3.31** | **[2.16; 5.07]** | **<0.001** | ******* |  | 1.30 | [0.87; 1.92] | 0.196 |  |
|  | Weekly SMS message | 1.37 | [0.96; 1.96] | 0.083 |  |  | **1.49** | **[1.06; 2.09]** | **0.021** | ***** |
|  | Monthly phone call from clinic | **2.65** | **[1.49; 4.73]** | **0.001** | ******* |  | 1.16 | [0.79; 1.71] | 0.440 |  |
| **Interaction effects** |  |  |  |  |  |  |  |  |  |  |
| Used PrEP x Information online |  | 0.92 | [0.53; 1.62] | 0.781 |  |  |  |  |  |  |
| Used PrEP x Start PrEP at community site |  | 1.18 | [0.76; 1.84] | 0.468 |  |  |  |  |  |  |
| Used PrEP x 6 monthly appointments |  | 0.90 | [0.43; 1.88] | 0.774 |  |  |  |  |  |  |
| Used PrEP x Collect PrEP at private pharmacy |  | 1.09 | [0.64; 1.87] | 0.748 |  |  |  |  |  |  |
| Used PrEP x PrEP delivery at home |  | 0.83 | [0.49; 1.42] | 0.501 |  |  |  |  |  |  |
| Used PrEP x HIV self testing |  | 0.75 | [0.41; 1.39] | 0.365 |  |  |  |  |  |  |
| Used PrEP x WhatsApp contact |  | 1.37 | [0.88; 2.15] | 0.168 |  |  |  |  |  |  |
| Used PrEP x SMS contact |  | 1.05 | [0.7; 1.58] | 0.815 |  |  |  |  |  |  |
| Used PrEP x Phone contact |  | 1.11 | [0.71; 1.74] | 0.648 |  |  |  |  |  |  |
| Scale (Tau) |  | 0.0898 | [-0.17; 0.34] | 0.4905 |  |  |  |  |  |  |
| n | 2456 |  |  |  |  |  |  |  |  |  |
| LL | -1755.6 |  |  |  |  |  |  |  |  |  |
| Pseudo R2 | 0.349 |  |  |  |  |  |  |  |  |  |

*****: p<0.05, ******: p<0.01, *******: p<0.001
